# Supplementary material for: Splice-Junction-Based Mapping of Alternative Isoforms in the Human Proteome
Source: Cell Rep. Author manuscript; Available in PMC 2020 Jan 15. (PMC6961840; doi:10.1016/j.celrep.2019.11.026)
Supplement: 2 [file NIHMS1546469-supplement-2.zip › DF1/PXD006675/LeftAtrium/LeftAtrium_43_FHL1_GSSVVAYEGQSWHDYCFHCKKCSVCR.pdf]

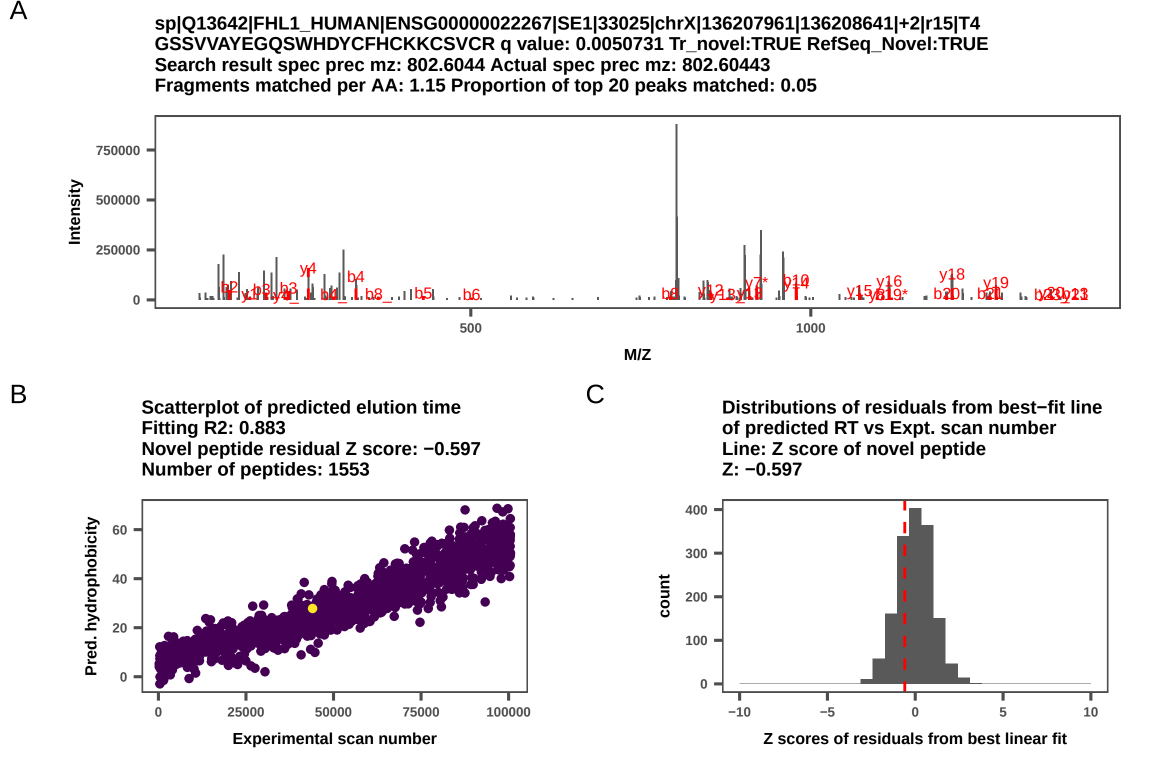
A
sp|Q13642|FHL1_HUMAN|ENSG00000022267|SE1|33025|chrX|136207961|136208641|+2|r15|T4
GSSVVAYEGQSWHDYCFHCKKCSVCR q value: 0.0050731 Tr_novel:TRUE RefSeq_Novel:TRUE
Search result spec prec mz: 802.6044 Actual spec prec mz: 802.60443
Fragments matched per AA: 1.15 Proportion of top 20 peaks matched: 0.05
Intensity
750000
500000
250000
0
500
1000
M/Z
B
Scatterplot of predicted elution time
Fitting R2: 0.883
Novel peptide residual Z score: −0.597
Number of peptides: 1553
Pred. hydrophobicity
60
40
20
0
0
25000
50000
75000
100000
Experimental scan number
C
Distributions of residuals from best−fit line
of predicted RT vs Expt. scan number
Line: Z score of novel peptide
Z: −0.597
count
400
300
200
100
0
−10
−5
0
5
10
Z scores of residuals from best linear fit
